# Strengthening pharmaceutical systems for palliative care services in resource limited settings: piloting a mHealth application across a rural and urban setting in Uganda

**DOI:** 10.1186/s12904-016-0092-9

**Published:** 2016-02-19

**Authors:** Eve Namisango, Chris Ntege, Emmanuel B. K. Luyirika, Fatia Kiyange, Matthew J. Allsop

**Affiliations:** African Palliative Care Association, Kampala, Uganda; Hospice Africa Uganda, Kampala, Uganda; Academic Unit of Palliative Care, Leeds Institute of Health Sciences, Faculty of Medicine and Health, University of Leeds, Leeds, UK

**Keywords:** Palliative care, Pharmacy, mHealth, Electronic system, Morphine, Pain management

## Abstract

**Background:**

Medicine availability is improving in sub-Saharan Africa for palliative care services. There is a need to develop strong and sustainable pharmaceutical systems to enhance the proper management of palliative care medicines, some of which are controlled. One approach to addressing these needs is the use of mobile technology to support data capture, storage and retrieval. Utilizing mobile technology in healthcare (mHealth) has recently been highlighted as an approach to enhancing palliative care services but development is at an early stage.

**Methods:**

An electronic application was implemented as part of palliative care services at two settings in Uganda; a rural hospital and an urban hospice. Measures of the completeness of data capture, time efficiency of activities and medicines stock and waste management were taken pre- and post-implementation to identify changes to practice arising from the introduction of the application.

**Results:**

Improvements in all measures were identified at both sites. The application supported the registration and management of 455 patients and a total of 565 consultations. Improvements in both time efficiency and medicines management were noted. Time taken to collect and report pharmaceuticals data was reduced from 7 days to 30 min and 10 days to 1 h at the urban hospice and rural hospital respectively. Stock expiration reduced from 3 to 0.5 % at the urban hospice and from 58 to 0 % at the rural hospital. Additional observations relating to the use of the application across the two sites are reported.

**Conclusions:**

A mHealth approach adopted in this study was shown to improve existing processes for patient record management, pharmacy forecasting and supply planning, procurement, and distribution of essential health commodities for palliative care services. An important next step will be to identify where and how such mHealth approaches can be implemented more widely to improve pharmaceutical systems for palliative care services in resource limited settings.

## Background

Effective analgesia and the availability of opioids are essential to support the range of holistic needs of patients who are in severe pain and have a terminal prognosis [1]. Access to medicines is a recognized and well-established universal human right; but one that is far from being guaranteed for people living in Africa [2]. In sub-Saharan Africa (SSA), many countries have restricted opioid formulations and access has been significantly impaired by widespread over-regulation, supply chain management and weak pharmaceutical systems [3–5]. African governments have taken critical steps to address issues of lack of access to pain management medication for patients living with terminal or life limiting illnesses. In April 2013, Ministers of health from the African Union agreed on a common position regarding palliative care and the use of opioids focusing on greater access and accountability. In September 2013, Ministers of health from 21 African countries adopted a “Consensus statement for palliative care integration into health systems in Africa: “Palliative Care for Africa” [6]. This statement outlines six objectives that are critical for integrating palliative care in health systems. Governments in the region are increasingly promoting policy level initiatives to integrate pain management and palliative care into existing health systems. Amidst these developments, it is essential to pursue concurrent development of effective and efficient pharmaceutical systems that can evolve and be adopted by countries with opioid availability. Approaches that ensure medicine stocks are well managed, reporting is done on time and ordering of stock is done on schedule can enhance service delivery [7].

While advocacy work persists to address regulatory barriers, there is a need to consider innovative approaches for achieving access to palliative care medicines. Innovation is critical given the urgent need to accommodate opioid provision within health systems as countries continue to integrate palliative care using the public health approach. As medicines are becoming available hospice and palliative care services need to develop strong and sustainable pharmaceutical systems to enhance the proper management of palliative care medicines, some of which are controlled. For example, in Uganda, a country applauded for improved availability of morphine [8], there persist major difficulties ensuring uninterrupted availability of morphine for the management of mild to severe pain. A study of 120 health facilities in Kenya and Uganda showed only 7 % of the health facilities (including 1 of 14 hospitals and 2 of 19 district hospitals) had access to morphine [4]. Notably, even in facilities with access to opioids, several service levels issues remained. Issues of stock-outs (i.e. no medicines available) were common and expiration of medications was noted. This complex situation calls for a comprehensive approach that will support adequate regulation and monitoring of increasing numbers of morphine prescribers across the country, alongside monitoring the supply chain mechanisms.

With the need to enhance provision of opioids and improve pharmaceutical management of pain medication in low resource settings, the African Palliative Care Association (APCA) sought to pilot an innovative approach to improving service delivery. Those involved in the distribution of the opioids at health facility level need enhanced logistics management skills and tools to support recording of quantities received, quantities dispensed and monitoring of stock levels, alongside a close watch for stock outs and expiries. One approach to address these needs is the use of mobile technology to support data capture, storage and retrieval. Utilizing mobile phone technology has been shown to enhance palliative care services through, for example, rapid access to clinical and social support networks and increasing health communication to patients and their caregivers [9]. mHealth refers to medical and public health practice supported by portable devices, such as mobile phones, patient monitoring devices, personal digital assistants, and other wireless devices [10]. Approaches using mHealth have demonstrated the capture of individual and health-related data at low cost, [11] and have been successfully used by frontline health care workers for remote data collection, remote monitoring, and diagnostic and treatment support in several developing countries [12]. Of particular advantage in low-resource settings are the fewer requirements on infrastructure compared to health information systems, making scale-up seemingly more feasible [13]. A mHealth approach was adopted to explore ways of strengthening the supply chain and service delivery components of palliative care pharmaceutical systems. This study reports on the piloting of an electronic pharmaceutical management application, accessed via tablet computers, to capture key patient and medicines data as part of opioids management in palliative care services.

## Methods

In this study we implemented an electronic application into palliative care services at two settings. By taking measures prior to implementation and identifying the changes to practice arising from introduction of the application, we investigated whether an electronic application can be used to replace hard copy documentation of routine reporting activities in palliative care clinics and associated pharmacies. The contribution of the application in supporting routine reporting of medicines receipt and dispensing was also explored. Ethical approval to undertake the study was obtained from Mildmay Uganda Research and Ethics Committee [REC Ref 0311–2015] and the protocol was approved and registered with the Uganda National council for Science and Technology [Ref HS 1972].

### Study setting

The study was conducted at two health facilities; an urban hospice and a rural district hospital. The rural setting, Hospice Africa Uganda, is located in a Kampala suburb with an average monthly patient population of around 800 patients, offering control of pain and symptoms during critical illness and at the end of life. This is combined with a patient-centered holistic approach to the patient and their family. The rural setting, Gombe district hospital, has an average monthly outpatient attendance of 2000 patients, with around 150 patients with palliative care needs. APCA and its partners have previously supported the integration of palliative care into routine patient care at Gombe hospital where palliative care is currently offered as part of the mainstream clinical care services. For the pilot at Gombe hospital, the intervention specifically targeted strengthening pharmaceutical systems for palliative care medicines as opposed to the entire hospital. The inclusion of Gombe hospital was important to explore integration of a mHealth intervention into a public health facility. Such facilities have been shown to be significantly worse than private hospitals at ensuring uninterrupted medication supplies [14].

### Overview of the application architecture

**Fig. 1:**
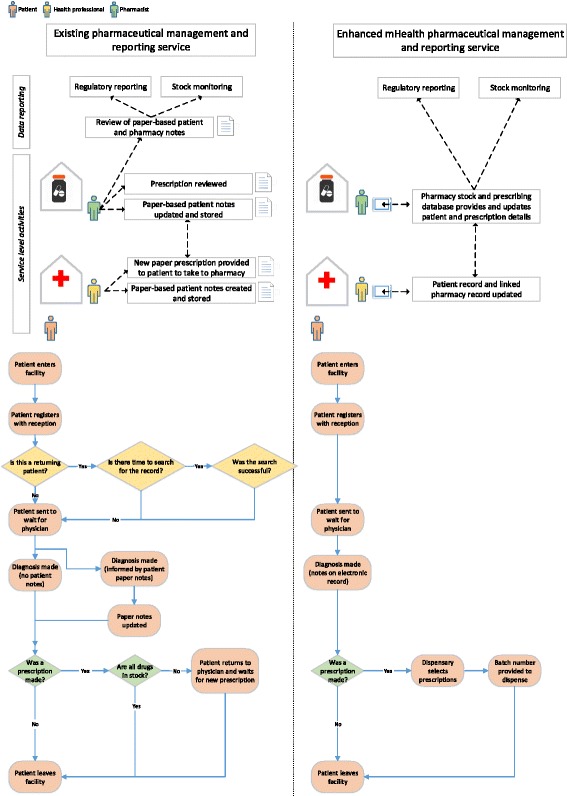
Overview of existing flow of information in service alongside enhanced model utilizing mHealth to consolidate multiple paper-based activities

A software application was designed to replace hard copy documentation of activities in palliative care pharmacies alongside facilitating routine reporting of medicines receipt and dispensing. The term ‘application’ refers throughout to the end user content that was accessed by health professionals. Figure 1 depicts planned changes to service processes from the introduction of the application in the pilot study. At a health systems level, the application was developed to target a number of system challenges and constraints. A visual framework, detailing mHealth innovations as health system strengthening tools [15], sought to provide a shared language around the description of mHealth applications. The application developed and described in this study targets four existing categories of use outlined on the visual framework; data collection and reporting, electronic health records, provider work planning and scheduling, and supply chain management.

The application was developed from a platform initially developed for use in a community health centre in Kampala that had proven robust in data transfer and storage. The application is designed for access by a mobile technology device, such as a tablet computer or a mobile phone. To inform the design of the content and layout of the application, an expert in pharmaceutical systems (author CN) led engagement activities with members of staff who were likely end users (nurses, doctors, pharmacists and dispensers) at the two site settings. The application was designed to support electronic patient record creation to enable easier documentation and retrieval of their data by health professionals. The electronic format also introduces more advanced approaches to protecting patient confidentiality. The application was also designed to comply with regulatory requirements while improving the efficiency of surrounding processes (data entry, reporting on prescriptions and medicine stock levels). In Uganda, there are three key documents used to capture data on narcotics and psychotropic medicines that must be in every pharmacy that dispenses Prescription Only Medicines: the Pharmacy Class A Medicines Register Book (a pharmacy ledger and requisition and issue voucher), quarterly returns form for class A medicines book (documenting issue and receipt of morphine or class A medicines, location of health unit and number of patients and their conditions), and ward issue voucher (issuing of class A medicines to patients on wards). The storage of hard copies of data means that operational tasks, such as medicine reports and review of patient notes can be time-consuming and burdensome. The application was tailored to capture these data electronically as there are currently no existing electronic platforms to support pharmacies supporting palliative care delivery in SSA.

**Fig. 2:**
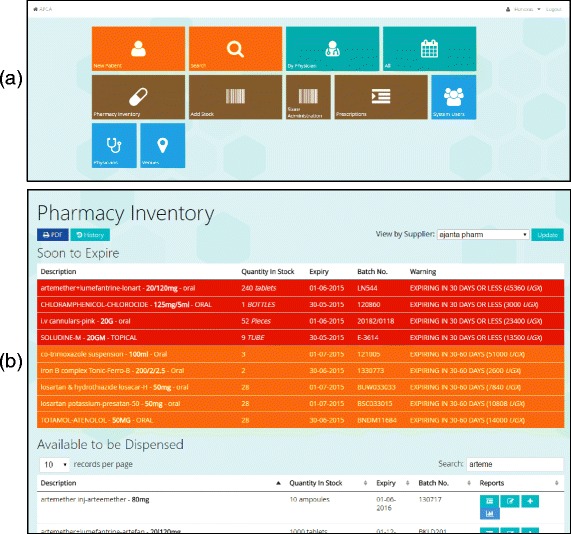
Examples of application interface with (a) view of dashboard as seen by health professionals: tabs are available for entering patient and pharmacy data, and (b) an example of inventory page in the application to highlight soon-to-expire medication

Continued engagement with staff throughout development ensured that the software was developed to align with existing working practices and improve existing service provision. For example, it was noted that often patients are prescribed medication during a consultation, arrive at the dispensary and are sent back to clinic because the medication is out of stock. A drug availability screen was developed in the application that enabled doctors to review all available drugs on one page during consultations to try to prevent this occurring. Additionally, patients could potentially arrive at the dispensary with paper prescriptions that were not recorded in the application. A prescription-making function was added to the application to enable the dispensary to log the patients and link them to stock information. User engagement also enabled refinement of the dashboard seen when first accessing the application (see Fig. 2a), such as colour-coding the functions by patient-related tasks, medicines stock and inventory, and system administrative activities.

### Application data collection

The application is used to capture data as part of routine care at both the patient and service level.

For each patient, data could be collected on:

- ♦ Socio-demographic information (age and sex)
- ♦ Major complaints and symptoms
- ♦ Diagnosis
- ♦ Pain rating on a 0 – 5 scale (if patient reports pain)
- ♦ Laxative prescription record (if patient receives opioid)
- ♦ Medical prescription
- ♦ Date of next appointment
- ♦ Phone contact details

Supply chain and service delivery information captured included:

- ♦ New stock of medication (including name, batch number, formulation, source, quantity and date of expiry)
- ♦ Quantities of palliative care medicines dispensed
- ♦ Identity of staff prescribing opioids
- ♦ Contact of medical personnel
- ♦ Frequency of stock outs

The software supports data analysis functions, such as comparing prescriptions and pain ratings across different conditions. Drug information captured by the application included history of the drugs dispensed, batch number and expiry date recording, and dispense instructions (ordered by expiry date) to minimize expirations. Dispensing instructions are enhanced by notifications and red highlighting of soon-to-expire medicine to clearly identify stock on the application inventory page (See Fig. 2b). All data captured by the application was stored on an off-site server. A virtual, secure network system was created with a live, password-protected link to the databases to enable technical study staff to access the data.

### Equipment

To access the application, each health facility was given two tablet computers (Samsung Galaxy Tab 3 (v8.0, 4G) with 16 GB storage). Electronic and solar power banks and portable power pack (Move Power Bank, Move Power 100,000mAh, input: 5 V 1A, output 1: 5 V 1A, output 2: 5 V 2A) and keyboards were provided for both tablets. Nurses, doctors, pharmacists and dispensers at each site had access to the tablets to enter all patient and medicines data. All tablets had internet connection which was renewed on a monthly basis.

### Training and technical support

At both sites, a one-day face-to-face training session was offered to study staff. The training included orientation on the application and basics for troubleshooting. This was followed by a series of four visits to each site at the start of the study to support setup and implementation in routine practice. One champion user who had technical knowledge in using electronic pharmaceutical systems at national level (author CN) provided basic support to staff at the two sites in case of any technical issues. He also gave guidance on how to modify the application to meet requirements of palliative care pharmaceutical systems. This was done through an iterative process where critical paths that could not be addressed by the system were outlined to the developer. The system would be modified and feedback received from potential users. Key issues included the capability to include batch numbers for medicines, catering for different strengths of oral morphine, enabling pain reports to be submitted, adding an option for documenting where medicines are prescribed but are out of stock, and sending automated reminders to the pharmacy manager about medicines that are about to expire. All tablets used in the study also had access to a messaging application that enabled users to alert the main software developer of technical issues that required attention.

### Outcomes

Table 1 outlines the indicators that were assessed at baseline and at six months post-implementation. These were developed around the intended purpose of the application: to replace hard copy documentation of activities in palliative care pharmacies alongside facilitating routine reporting of medicines receipt and dispensing.

**Table 1:** Indicators to assess strengthening of palliative care pharmaceutical application

Metric	Outcome	Measure
Completeness of data capture	*Entry of data for key patient variables*	Proportion of patient entries to application with sociodemographic data, diagnosis, complaints, symptoms and contact details
	*Patient prescription recording*	Documentation of prescriptions to accompany patient records
Time efficiency	*Patient medical record retrieval*	Time elapsed from patient arrival at the information or triage centre to when a patient file is located and referred to the next level to start the care process
	*Time spent on ordering stock*	Time required to take stock, calculate consumption rates and then determine quantities to order
	*Time spent on preparing pharmacy reports*	Time spent on establishing stock arrival and dispensing, the value of existing stock and variances between physical stock count and stock card value
Medicines stock and waste management	*Frequency of stock outs for opioids*	Frequency of emergency orders logged on application
	*Percentage of whole stock value that expired per quarter*	Proportion of stock that expired within a quarter from total stock

### Data collection for targeted indicators

Data collection sought to understand both the extent of use and completeness of data captured by the application alongside the impact of the application on practice. Completeness of data capture was assessed by examining the extent of documentation for each new patient entry and looking at whether all fields had been completed. Time efficiency metrics were assessed by research team members at each site. Activities (record retrieval, time to order stock, report preparation) were timed at baseline and after 6 months, by taking an average from across 10 timed patient visits. In addition, technical issues arising during the implementation of the application were logged by the champion user who supported technical issues (author CN). Data analysis involved descriptive reporting of measures sought at the start of the study and after 6 months. Patient identifiers were removed from the database prior to any analysis and access was only granted via a password protected link.

## Results

The application was implemented into routine practice across two sites, with reporting performed for data completeness, time efficiency and medicine stock levels. There are also added observations by the research team around implementation.

### Completeness of data capture

Data completeness was assessed to explore whether the application could support the generation of well documented management plans of care for patients in an electronic format for easy and fast access.

#### Entry of data for key patient variables

The application was used to register patients (*n* = 455; 238 females) across two sites; both the hospice (*n* = 381) and rural clinic (*n* = 74; 36 females). When compared to the figures of all palliative care patients managed by the two sites at the time of the study, electronic patient records had been established for 47 % (381/807) of the hospice patients and 47 % (74/157) of patients at the rural clinic. The median age of patients with electronic records established was 52 years (range of 93 (3–96 years)) at the hospice site and 38 years (range = 80 (5 – 85 years)) at the rural clinic.

As shown in Table 2, the period of data collection differed between the sites, with a longer period of time dedicated to data collection at the rural clinic. In total, 565 consultations were recorded from both sites, with 309 prescriptions recorded. The majority of patients had one consultation, but there was documentation of multiple visits for some patients across both sites. Pain scores were captured for 12 % of consultations at the hospice site and 78 % of consultations at the rural site. Mobile phone contact details were recorded for a patient or caregiver for 18 % of patients at the hospice and 55 % of patients in the rural clinic.

**Table 2:** Overview of recorded consultations and prescriptions at both sites

	Urban hospice	Rural clinic
*Patient data capture*		
Time period of data collection	197 days (Sep 14 – Mar 15)	297 days (Aug 14 – May 15)
Number of patients recorded on application	*n* = 381	*n* = 74
Number of consultations recorded	*n* = 474	*n* = 88
*Number of patients with 1 consultation*	*n = 314*	*n = 60*
*Number of patients with 2 consultations*	*n = 44*	*n = 13*
*Number of patients with 3 consultations*	*n = 18*	*n = 1*
*Number of patients with 4 consultations*	*n = 5*	*n = 0*
Median initial pain score (1 - 5)	3 (*n* = 57; 12 % of consultations)	3 (*n* = 69; 78 % of consultations)
Mobile phone contact details recorded	*n* = 68 (18 % of patients)	*n* = 41 (55 % of patients)
*Prescribing data*		
Median number of prescriptions per consultation	*n* = 2(range = 8)	*n* = 0 (range = 4)
*Number of consultations with no prescription*	*n* = 68	*n* = 57
*Number of consultations with 1 drug prescribed*	*n* = 58	*n* = 12
*Number of consultations with 2 drugs prescribed*	*n* = 134	*n* = 14
*Number of consultations with 3 drugs prescribed*	*n* = 99	*n* = 3
*Number of consultations with >3 drugs prescribed*	*n* = 119	*n* = 2
*Number of individual drugs prescribed at site*	*n* = 474a	*n* = 57
*Morphine prescribing*		
Total morphine prescriptions (% of all consultations)	*n* = 281 (59.03 %)	*n* = 26 (29.21 %)
Morphine (oral or MST) only	*n* = 65 (13.66 %)	*n* = 11 (12.34 %)
Morphine and laxative	*n* = 216 (45.33 %)	*n* = 15 (16.85 %)
*Laxative only*	*n* = 23 (4.83 %)	*n* = 0 (0 %)
*Prescription(s) not including morphine or laxative*	*n* = 172 (36.13 %)	*n* = 62 (69.66 %)
Proportion of all morphine prescriptions with laxative	76.87 %	57.69 %
Proportion of all morphine prescriptions without laxative	23.13 %	42.31 %

#### Recorded prescriptions over time

Table 2 outlines the number of prescriptions recorded at both sites. There was a higher median number of prescriptions made at the hospice site. At the rural clinic, most consultations ended without a prescription being recorded.

Morphine prescriptions comprised over half of all prescriptions at the hospice, but nearer a third for the rural clinic. Of morphine prescriptions made, morphine alone was prescribed for 14 % of patients at the hospice and 12 % at the rural clinic. The proportion of all morphine prescriptions which included a laxative was higher at the hospice (77 %) than the rural clinic (58 %).

**Fig. 3:**
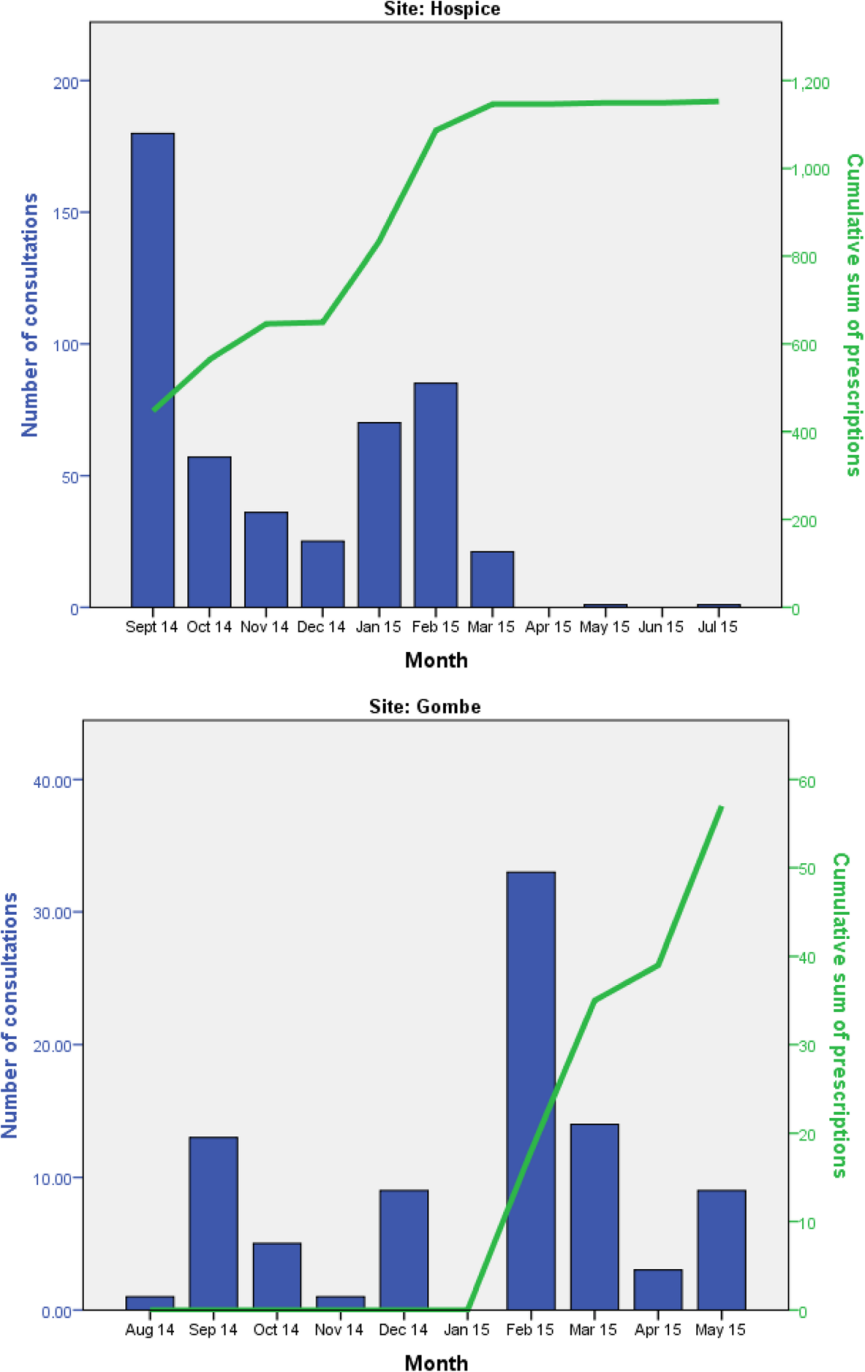
Graphs to show increased cumulative prescriptions over duration of study

Figure 3 partly explains lower figures recorded by the rural clinic site. The graph details consultations occurring at varying rates during the start of their involvement in the study, but with no prescriptions being filed. This may indicate an issue with using the application or the lack of prescriptions being filed for patients during this time. Registration of patients during the later stages of the hospice involvement in the study was also dramatically reduced.

### Time efficiency

The introduction on an electronic application was designed to reduce the time currently taken by pharmacy teams to carry out standard operational activities. These include retrieving medical records, ordering stock and preparing pharmacy reports. These tasks are aligned to regulatory requirements of pharmacies so may improve the efficiency of routine and necessary reporting activities.

#### Patient medical record retrieval, time spent ordering stock and time spent on preparing pharmacy reports

Table 3 outlines the changes in time dedicated to routine activities and reporting for the pharmacy teams at each site. For both sites, there were reductions in the time taken to retrieve patient records, time spent on ordering stock, and on time spent preparing pharmacy reports. Notably the time spent preparing pharmacy reports was reduced from 14 days to 1 at the hospice site and from 5 days to 2 days at the rural clinic.

**Table 3:** Before and after performance on selected indicators

	Urban hospice	Rural hospital
Time Efficiency		
*Time spent retrieving record*		
Baseline	30 min	45 min
6 months’ post-implementation	5 min	5 min
*Time spent on ordering stock*		
Baseline	7 days	10 days
6 months’ post-implementation	30 min	1 h
*Time spent on preparing pharmacy reports*		
Baseline	14 days	5 days
6 months’ post-implementation	1 day	2 days
Medicine stock and waste management		
	Urban hospice	Rural hospital
*Frequency of stock outs of opioids*		
Baseline	3 per quarter	2 per quarter
Post-implementation	1 per quarter	1 per quarter
*Percentage of whole stock value that expired per quarter*		
Baseline	3 %	58 %
Post-implementation	0.5 %	0 %

### Medicine stock and waste management

This process of medicine stock and waste management involves recording received and prescribed quantities, alongside tracking the stock value and variances between physical stock count and stock card value to explain differences.

#### Frequency of stock outs of opioids and percentage of whole stock value that expired per quarter

The frequency of stock outs reduced as ordering became more accurate and this dramatically reduced number of stock outs and emergency orders. This was measured by frequency of emergency orders, which reduced from five times a quarter to two times a quarter at the hospice site and from two times a quarter to one at the rural hospital.

### Additional observations made by research team

The research team noted issues that had to be considered when working across the urban and rural settings. The provision of solar energy packs was important for the users of the application in the rural setting, alongside having the mechanism for working offline during internet outages. Both power failures and internet outages were common in this setting.

The application can be used for managing common medications, but its use in a palliative care setting meant that medications were strictly focused on those typically used with this patient group. This was reported as particularly helpful to the rural hospital because medicines at their site are centrally stored and managed. Medications for use in palliative care include opioids, for which there are special reporting requirements and dedicated pharmaceutical micro-management plans required. Limiting medications on the system for the rural hospital supported stock management by making it more targeted for palliative care medicines, which made tracking and reporting easier.

Low reporting of contact details were identified at both sites, particularly the urban hospice. Causes identified by the research team include the lack of emphasis on mobile phone use in routine care, with a strong home-based care programme in place at the urban hospice. Patients failing to attend a hospice outpatient appointment typically receive a visit from a community team. For the rural hospital, at the time of the study integration of palliative care into routine care had only just taken place, with plans for detailing patient contact details (e.g. for use in appointment reminder text messages) not prioritized at the time.

While the iterative development of the system promoted a dynamic, bottom-up approach to design, it skewed study data for pain rating collection. There are differences across the sites in the proportion of consultations where a pain rating was documented. The capability to add pain ratings was only enabled in January 2015, with the rural hospital having a longer period of data collection with this field active. This may, in the most part, explain the higher proportion of pain reports in this setting. The minor adjustments to the application during the study period were necessary for clinical use and are a consequence of adopting a pragmatic approach to piloting and evaluation of the application.

## Discussion

This study reports on the piloting of an electronic pharmaceutical management application in an urban and a rural setting. The application was adopted by healthcare professionals who used the application to replace hard copy documentation of routine reporting activities completed for palliative care patients and their medications. Comprehensive capture of nearly half of all patients accessing care at the facilities, reductions in the time taken to complete routine documentation and reporting for pharmacy staff, and reductions in stock waste were measured. The research team also observed a number of factors to consider which may inform the future development of the application. Currently, most palliative care facilities mainly operate using a manual paper-based inventory system, yet they have a large inventory of items to procure, manage and report on. As a result, it is common to experience unnecessary stock losses due to expiry of pharmaceuticals which could be better managed with the use of a computerized inventory management application. Our results show that the frequency of stock outs reduced as ordering was being made accurately and in time and this significantly reduced the number of stock outs and emergency orders. Given the high prevalence of stock outs for opioids commonly cited in palliative care, a mHealth approach is of great value as it assists with mapping health facilities without opioids, those with low stock levels and those with stock that is about to expire. In latter instances, having this information could direct efforts to transfer stock to health facilities that can use it promptly and reduce on resource wastage.

Refining pharmaceutical reporting in palliative care is essential. Current weaknesses in reporting have an impact on opioid availability at the national level as many countries fail to submit estimates for their need for controlled substances based upon careful assessment of population needs to the INCB, as required by the UN drug conventions. Indeed, many other countries submit estimates that vastly understate the actual medical need for morphine and cannot ensure adequate availability of opioids for pain treatment in their respective countries. Often these estimates are not based on actual need but on morphine consumption during the previous year. The source for morbidity data is the health facility and if data on consumption by type of diagnosis is captured accurately, it makes it easier for those involved in estimation and ordering to compute and submit accurate needs-based quantities for consumption. The availability of consumption data will also improve the accuracy of the quantification of medicines, decreasing the risk of stock-outs and expiries.

This study identifies benefits that can be derived from mHealth approaches to directly enhance the operational processes of clinical and pharmacy services in palliative care. In particular, it presents a low cost and efficient approach to facilitating pharmaceutical management. Globally there remain major country-level disparities in access to opioids for pain relief at end of life [16], with a key barrier being a lack of supply and distribution systems. A goal stemming from a World Innovation Summit for Health is the need to ensure that essential medicines are nationally available in central medical stores, licensed appropriately, and distributed effectively through existing health services [17]. With mobile technology capable of supporting tasks such as automation [18], data collection [19] and operations, there is an opportunity to strengthen pharmaceutical management while additionally mapping the burden of pain and requirements for opioids in palliative care patients. Technology has the ability to facilitate empathic patient-professional relationships in the support of advanced disease in both high, middle and low income settings [20, 21]. Continuing to chart lessons from the implementation of mobile technology in palliative care has relevance across services worldwide. This study highlights an approach that utilized technology to improve pharmaceutical reporting in response to both legislative and regulatory requirements. Exploring approaches that leverage mobile technology to strengthen pharmaceutical systems in palliative care has global relevance; barriers and restrictions to effective opioid provision are reported widely across high and LMIC settings [22, 23].

A pilot study is a requisite initial step in exploring innovative approaches and interventions. While the implementation of the application seemed feasible across the sites, only around half of all patients accessing care at the facilities were registered. And for patients recorded on the application, certain fields had relatively low levels of completion (such as contact details and pain score reporting where pain is present). Future development should explore approaches to improving registration and recording of data for patients to inform the planning and design of a larger efficacy trial. In addition, improving the efficiency and ease of capturing patient reported outcomes, such as pain, are of importance to increase understanding of the effects and outcomes of care on patients. Refinement of the application can be informed by this study and, importantly, is provided as open-source software (OSS). OSS enables adaptation of applications for a local context and is possible with scarce economic resources.

The use of electronic systems to capture patient reported outcomes is an emerging research field. Capture of patient reported outcomes (PROMs) using mHealth applications deliver benefits including improved communication between patient and physician, improved patient satisfaction with care, and time savings [24]. PROMs are an important component to palliative care development in Africa [25], and understanding how they can be utilized through mHealth approaches could be a significant contribution to the field. This study suggests that mHealth approaches can be feasible options for supporting health professionals in the delivery of palliative care in SSA. However, mobile phone details were documented on the records of numerous patients. The potential of mHealth interventions for palliative care patients in SSA has been outlined in the literature, but research is required to identify how such interventions might be designed and developed [9].

## Conclusion

Approaches are desperately required to strengthen pharmaceutical systems in palliative care in SSA. A mHealth approach adopted in this study was shown to improve existing processes for patient record creation, pharmacy forecasting and supply planning, procurement, and distribution of essential health commodities for palliative care services. A move away from hardcopy documentation provided benefits in patient data security and reduced the time required for reporting. Through collecting and collating data electronically, pharmacies may be able to improve on medicine rational use, accountability, and reporting at both national and INCB level. mHealth approaches are at an early stage of development in SSA palliative care. An important next step will be to identify where and how such mHealth approaches can be implemented more widely to improve pharmaceutical systems for palliative care services in resource limited settings.
